# Mitigating the heroin crisis in Baltimore, MD, USA: a cost-benefit analysis of a hypothetical supervised injection facility

**DOI:** 10.1186/s12954-017-0153-2

**Published:** 2017-05-12

**Authors:** Amos Irwin, Ehsan Jozaghi, Brian W. Weir, Sean T. Allen, Andrew Lindsay, Susan G. Sherman

**Affiliations:** 1 Law Enforcement Action Partnership, Silver Spring, MD USA; 2 Criminal Justice Policy Foundation, Silver Spring, MD USA; 3 British Columbia Centre for Disease Control, University of British Columbia, Vancouver, Canada; 4 School of Population and Public Health, University of British Columbia, Baltimore, MD USA; 5 Department of Health, Behavior, and Society, Johns Hopkins University Bloomberg School of Public Health, Baltimore, MD USA; 6 Criminal Justice Policy Foundation, Amherst College, Silver Spring, MD USA

**Keywords:** Supervised injection facility, Supervised consumption rooms, Cost-benefit, Cost-effectiveness, People who inject drugs, Harm reduction, Opiate overdose, Heroin, Baltimore, Maryland

## Abstract

**Background:**

In Baltimore, MD, as in many cities throughout the USA, overdose rates are on the rise due to both the increase of prescription opioid abuse and that of fentanyl and other synthetic opioids in the drug market. Supervised injection facilities (SIFs) are a widely implemented public health intervention throughout the world, with 97 existing in 11 countries worldwide. Research has documented the public health, social, and economic benefits of SIFs, yet none exist in the USA. The purpose of this study is to model the health and financial costs and benefits of a hypothetical SIF in Baltimore.

**Methods:**

We estimate the benefits by utilizing local health data and data on the impact of existing SIFs in models for six outcomes: prevented human immunodeficiency virus transmission, Hepatitis C virus transmission, skin and soft-tissue infection, overdose mortality, and overdose-related medical care and increased medication-assisted treatment for opioid dependence.

**Results:**

We predict that for an annual cost of $1.8 million, a single SIF would generate $7.8 million in savings, preventing 3.7 HIV infections, 21 Hepatitis C infections, 374 days in the hospital for skin and soft-tissue infection, 5.9 overdose deaths, 108 overdose-related ambulance calls, 78 emergency room visits, and 27 hospitalizations, while bringing 121 additional people into treatment.

**Conclusions:**

We conclude that a SIF would be both extremely cost-effective and a significant public health and economic benefit to Baltimore City.

## Background

Baltimore City has one of the highest overdose death rates in the country, and overdoses have been increasing in recent years. From 2014 to 2015, heroin-related overdose deaths in Baltimore increased from 192 to 260 [1]. These increases are in part attributed to the prevalence of fentanyl in the heroin supply, with fentanyl causing 31 and 51% of 2015 and 2016 overdose deaths, respectively. Fentanyl is 50–100 times more potent than heroin or morphine. Illicit fentanyl and derivatives are appealing to illicit drug networks as these chemicals are cheaper than prescription opioids, heroin, and cocaine, and are extremely potent [2–5].

There are numerous additional medical costs associated with injection drug use, largely related to infectious diseases and soft-tissue infections. Roughly 18% of the people who inject drugs (PWID) in Baltimore are HIV positive, twice the 9% national average for PWID and 50 times the prevalence in the general population [6–8]. One in five Baltimore PWID suffers chronic skin and soft-tissue infection, the leading cause of PWID hospitalization [9–11].

Supervised injection facilities (SIFs) have been established worldwide to reduce the harms associated with injection drug use. In SIFs, PWID inject previously obtained drugs in the presence of medical staff. A number of public health, social, and economic benefits of SIFs have been evaluated by studies of the Insite SIF in Vancouver, Canada and the Medically Supervised Injecting Centre (MSIC) in Sydney, Australia, both of which were established in 2003 [12–15].

Among these benefits, studies have demonstrated four in particular that can be quantified. First, SIFs reduce blood-borne disease transmission by providing clean needles and safer injecting education [12, 16, 17]. Second, SIF staff reduce bacterial infection by providing clean injection equipment, cleaning wounds, and identifying serious infections early [18–20]. Third, SIF staff intervene in case of overdose, meaning that while PWID may overdose at a SIF, none die and few suffer complications [13]. Fourth, the SIF and its staff become a trusted, stabilizing force in many hard-to-reach PWID’s lives, persuading many to enter addiction treatment [12, 14, 21].

As in other US cities, a multisector discussion about the merits and utility of SIFs has begun in Baltimore due to rising overdose deaths as well as the inadequacy of the current criminal justice-focused response [22].

The purpose of this article is to analyze the potential cost-effectiveness of establishing a SIF in Baltimore. We estimate the annual cost of the facility and the savings resulting from six separate health outcomes: prevention of HIV infection, HCV infection, skin and soft-tissue infection (SSTI), overdose death, and nonfatal overdose and increased medication-assisted treatment (MAT) uptake. Each estimate includes the health outcome, financial value, and a sensitivity analysis. First, we present the existing literature on SIF cost-benefit analyses, then our study’s method, its results, its implications, and its limitations.

### SIF cost-benefit analysis literature review

Prior cost-benefit analyses of Insite in Vancouver and MSIC in Sydney have assessed a more limited range of outcomes than the present study. The Insite studies were limited to the outcomes of HIV prevention, HCV prevention, and overdose death prevention. They have agreed that Insite generates net savings when all three outcomes are considered [23, 24]. The cost-benefit analysis of Sydney’s MSIC only included savings from overdose deaths, ambulance calls, and police services averted by the SIF.

A number of other studies have estimated HIV and HCV prevention benefits for hypothetical SIFs in Canadian cities from Montreal to Saskatoon [25–30]. Irwin et al. [31] are the only other cost-benefit analysis of a hypothetical SIF in the USA—in San Francisco, California—and the only other study to consider more than three outcomes. We discuss the differences in methodologies between this paper and past analyses for each individual outcome in the “Methods” section.

## Methods

This study calculates the financial and health costs and benefits of a hypothetical Baltimore SIF modeled on Insite. Insite occupies roughly 1,000 ft^2^, provides 13 booths for clients, and operates 18 h per day. Insite serves about 2100 unique individuals per month, who perform roughly 180,000 injections per year [32, 33].

This study measures the cost of the facility against savings from six outcomes: prevention of HIV, HCV, SSTI, and overdose deaths, reduced overdose-related medical costs, and referrals to MAT. We assess each model’s dependence on important variables with a sensitivity analysis. For the sensitivity analysis, we increase and decrease the chosen variable by 50% and report the impact on the outcome.

### Cost of the facility

Cost calculations are based on a facility equal in size and scope to Insite. We estimate that the annual cost of establishing a new SIF combines both upfront and operating costs. Since we assume the same staffing levels, equipment needs, and other operating cost inputs as Insite, we calculate the operating costs by multiplying the Insite SIF’s $1.5 million operating costs by a 4% cost of living adjustment between Vancouver and Baltimore [34, 35]. Since the upfront costs would depend on the exact location and extent of renovations required, we make a conservative estimate of $1.5 million based on actual budgets for similar facilities and standard per-square-foot renovation costs [12, 36]. We convert this upfront cost into a levelized annual payment by assuming that it was financed with a loan lasting the lifetime of the facility. We determine the levelized annual payment according to the standard financial equation:

$$ C=\frac{i(P)}{1-{\left(1+ i\right)}^{- N}} $$

where *C* is the levelized annual upfront cost, *i* is a standard 10% interest rate, *P* is the $1.5 million total upfront cost, and *N* is the estimated 25-year lifetime of the facility.

### HIV and HCV prevention benefits

The HIV infection prevention benefits of Insite, Vancouver’s SIF, have been modeled in several cost-benefit analyses [23, 24, 37, 38]. Pinkerton [24] and Andresen and Jozaghi [23] estimate 5–6 and 22 infections averted per year, respectively. These estimates differ primarily because Pinkerton [24] assumes that the SIF only impacts injections occurring within the SIF, while Andresen and Jozaghi [23] incorporate the fact that the SIF reduces needle sharing outside the SIF as well, since Insite staff educate clients on safer injecting practices [38].

To estimate the impact of reduced needle sharing on HIV and HCV infection rates, we use an epidemiological “circulation theory” model developed to calculate how needle exchange programs impact HIV infection among PWID and subsequently used to study SIF HIV and HCV infection [23, 39]. We use the model to estimate new HIV infection cases (*I*_HIV_):

$$ {I}_{\mathrm{HIV}}= i N s d\kern0.21em \left[1-{\left(1- qt\right)}^M\right] $$

where *i* is the percentage of HIV-negative PWIDs, *N* is the total number of needles in circulation, *s* is the percentage of injections with a shared needle, *d* is the percentage of injections with an unbleached needle, *q* is the percentage of HIV-positive PWIDs, *t* is the chance of transmitting HIV through a single injection with a shared needle, and *M* is the average number of people injecting with a single previously used needle. Table 1 shows the values and sources for each variable.

**Table 1:** Values, notes, and sources for variables used to predict HIV infection reduction savings

Variable	Value	Note	Source
Proportion of PWID HIV− (*I*)	82.30%		Mehta [6]
Number of needles in circulation (*N*)	1,600,000	Increased by 33% due to additional syringe sources	Hunt and Parker [81];German et al. [82]
Rate of needle sharing (*s*)	2.8%	Percent of injections with a needle already used by another person	Park et al. [83]
Percentage of needles not bleached (*d*)	100%		Bluthenthal et al. [84]
Proportion of PWID HIV+ (*q*)	17.70%		Mehta [6]
Probability of HIV infections from a single injection (*t*)	0.67%		Kaplan and O’Keefe [85]; Kwon et al. [86]
Number of sharing partners (*m*)	1.2	Per injection: ratio of receptive to distributive sharing	Park et al. [83]
SIF client reduction in needle sharing (*n*)	70%	From Insite	Kerr et al. [40]
Number of SIF clients (*N*)	2100	Approximate monthly unique Insite injection room clients	Maynard [33]
PWID Population (*T*)	20,950	Adjusted by authors from Baltimore MSA to City using race census data	Tempalski et al. [87]
Lifetime HIV treatment cost	$402,000	National data	CDC [88]

We estimate SIF-averted HIV infections by finding the difference between *I*_HIV_ at the current rate of needle sharing (*s*_pre_) and *I*_HIV_ at the post-SIF rate (*s*_post_). We calculate *s*_post_ with the formula:

$$ {S}_{\mathrm{post}}={S}_{\mathrm{pre}}\frac{\left( T- N\right)+\left(1- n\right) N}{T} $$

where *T* is the total number of PWID in Baltimore City, *N* is the number of SIF users, and *n* is the 70% reduction in needle sharing by SIF users [40].

We perform the same calculations for HCV, and the values and sources for the HCV variables are contained in Table 2.

**Table 2:** Values, notes, and sources for variables used to predict HCV infection reduction savings

Variable	Value	Note	Source
Proportion of PWID HCV− (*I*)	25%	Average of values reported (10–40%)	Falade-Nwulia et al. [89]
Number of needles in circulation (*N*)	1,600,000	Increased by 33% due to additional syringe sources	Hunt and Parker [81]; German et al. [4]
Rate of needle sharing (*s*)	2.8%	Percent of injections with a shared needle	Park et al. [83]
Percentage of needles not bleached (*d*)	100%		Bluthenthal et al. [84]
Proportion of PWID HCV+ (*q*)	75%	Average of values reported (60–90%)	Falade-Nwulia et al. [89]
Probability of HCV infections from a single injection (*t*)	3%		Kwon et al. [86]; Kaplan and O’Keefe [85]
Number of sharing partners (*m*)	1.2	Per injection: ratio of receptive to distributive sharing	Park et al. [83]
SIF client reduction in needle-sharing (*n*)	70%	From Insite	Kerr et al. [40]
Number of SIF clients (*N*)	2100	Approximate monthly unique Insite injection room clients	Maynard [33]
Total PWID population (*T*)	20,950	Adjusted by authors from Baltimore MSA to City using race census data	Tempalski et al. [87]
Lifetime HCV treatment cost	$68,219	Adjusted for inflation	Razavi et al. [90]

We check the model’s validity by comparing its baseline prediction of HIV and HCV incidence in Baltimore (*I*_HIV_ and *I*_HCV_ at *s*_pre_) with the city’s actual incidence data. The model predicts 53 new PWID-related HIV cases in Baltimore each year in the absence of a SIF, only slightly lower than the 55 diagnoses reported by the Maryland Department of Health and Mental Hygiene [41]. Since many new HIV cases go undiagnosed, especially in the hard-to-reach PWID population, this baseline figure suggests that we are underestimating potential HIV infections averted [42].

For HCV, the model predicts 302 cases in the absence of a SIF. The Maryland Department of Health and Mental Hygiene (DHMH) does not report annual injection-related HCV infections for Baltimore City. However, based on Mehta et al.’s [43] finding that 7.8% of a sample of Baltimore’s HCV-negative PWID contract HCV every year, we estimate PWID HCV incidence at 398 new cases per year. Since our model predicts a significantly lower incidence, we are most likely underestimating the potential number of HCV infections averted.

### Skin and soft-tissue infection benefits

Since PWID frequently contract skin and soft-tissue infection from unsanitary injection practices and often avoid seeking medical treatment until these infections become life threatening, SSTI is the number one reason for PWID hospital admission. Insite studies have demonstrated that SIFs significantly reduce SSTI medical costs by providing clean injection materials and referring PWID for medical treatment when necessary [18, 20]. Irwin et al. [31], the only cost-benefit analysis to incorporate this outcome, have shown this outcome to be significant, concluding that a SIF in San Francisco could reduce SSTI-related hospitalizations by 415 days per year, saving $1.7 million.

We estimate annual savings due to SIF SSTI reduction (*S*_SSTI_) according to

$$ {S}_{\mathrm{SSTI}}= NhLrC $$

where *N* is the total number of SIF clients, *h* is the percent of PWID hospitalized for SSTI in an average year, *L* is the average length of SSTI hospitalization, *r* is the 67% reduction in SSTI hospital stay length that Lloyd-Smith et al. [18] documented for Insite clients, and *C* is the average daily cost of a hospital stay. See Table 3 for values and sources.

**Table 3:** Values, notes, and sources for variables used to predict skin and soft-tissue infection reduction savings

Variable	Value	Note	Source
Number of SIF clients (*N*)	2100	Approximate monthly unique Insite injection room clients	Maynard [33]
Hospitalization rate for skin and soft-tissue infection (*h*)	4.43%	Includes abscesses, cellulitis, sepsis, endocarditis, septic arthritis, osteomyelitis	Hsieh [91]; Lloyd-Smith et al. [18]; Kerr et al. [92]
Reduction in soft-tissue and skin infection for PWID that visit SIF (*r*)	67.00%	From Insite	Lloyd-Smith et al. [18]
Average length of skin infection-related hospital stay for PWID (*L*)	6 days	From Baltimore (Hsieh, 2015)	Hsieh [91]; Lloyd-Smith et al. [18]; Stein [93]; Palepu et al. [94]
Average hospital cost per day (*C*)	$2500	Average cost per inpatient day, not specifically for PWID	Rosenthal [95]; Harris [96]

### Overdose mortality benefits

While Andresen and Boyd [44] estimate that Insite prevents one overdose death per year, out of roughly 20 total overdose deaths in the neighborhood, they are simply extrapolating that if Insite hosts 5% of the city’s injections, it should prevent 5% of the city’s overdose deaths. However, Milloy et al. [45] demonstrate that Insite prevents more than 5% of the city’s overdose deaths. Milloy et al. attribute this effect to drug use education, which 32% of all Insite clients report receiving. For example, PWID learn to pre-inject a small dose of their drug to “test” the potency, which can prevent accidental overdose in case of an unusually pure or contaminated dose. In Sydney’s SIF, known as MSIC, 80% of clients report changing their injection behavior to reduce the risk of overdose as a result of in-SIF education [15].

This finding is supported by Marshall et al. [46], who compare the change in overdose deaths within 500 m of Insite to the change in other Vancouver neighborhoods both before and after the facility’s opening. They find that before Insite opened, roughly 20 overdoses occurred within 500 m of the facility. After Insite opened, overdose mortality within 500 m of the facility fell by 35%, compared to a 9.3% reduction further away, suggesting that Insite reduced neighborhood overdose deaths by at least 26% [46].

Therefore, to predict the impact of a SIF on fatal overdose, we estimate the number of overdose deaths within a 500-m radius of an optimally placed SIF in Baltimore. Based on the fact that there were 260 heroin-related fatal overdoses in 2015 and 342 in the first three quarters of 2016, we estimate that there were 463 heroin-related fatal overdoses in all of 2016 [1, 47]. Since data on the geospatial distribution of fatal overdoses in Baltimore City are not available, we approximate this distribution by mapping data from the Baltimore City Fire Department Emergency Medical Services on the locations where medics administered naloxone in response to suspected opioid overdoses [48]. We identify the location with the highest concentration of naloxone administrations within 500 m by plotting the locations of all naloxone administrations in the first three quarters of 2016 in ArcGIS. The chosen location accounts for 6.2% of all naloxone administrations, suggesting that 28 heroin-related overdose deaths occurred within that 500-m radius circle in 2016. As the percent of overdose deaths within this area varies over time, we assume that in an average year, it would encompass a more conservative 23 heroin-related overdose deaths. This is 5% of the city-wide total and slightly higher than the 20 deaths per year within 500 m of Insite.

We calculate the total value of overdose deaths averted by the SIF (*S*_OD_) according to the equation:

$$ {S}_{\mathrm{OD}}= rnDV $$

where *r* is the rate of overdose death reduction expected within 500 m, *n* is the 5% share of naloxone administrations concentrated within a single circle of radius 500 m in Baltimore, *D* is the total number of overdose deaths in Baltimore, and *V* is the value of a single life saved.

In order to assign value to the loss of life due to overdose, we follow Andresen and Boyd [44] in considering only the tangible value to society rather than including the suffering and lost quality of life for loved ones. We estimate the tangible value by calculating the present value of the remaining lifetime wages of an average person from the community. Since the average age of PWID in Baltimore is 35, we convert 30 years of future wages to present value using a standard discount rate [44, 49]. So the value of a single prevented overdose death (*V*) is calculated as

$$ V={\displaystyle \sum_{i=1}^N\frac{W}{{\left(1+ r\right)}^i}} $$

where *n* represents the remaining years of income, *W* represents the median wage for Baltimore City, and *r* represents the discount rate. We thus use a value per life saved of $503,869 in the overdose death savings calculation above. The values and sources for each variable in this section are given in Table 4.

**Table 4:** Values, notes and sources for variables used to predict savings from averted overdose deaths

Variable	Value	Note	Source
Percent overdose death reduction within 500 m attributed to Insite (*r*)	25.7%	36% reduction within 500 m of Insite vs. 9.3% further away	Marshall et al. [46]
Largest share of naloxone administrations within 500-m radius in Baltimore (n)	5%	Lowered from 6.2% to account for reversion to mean based on limited years of data	BCFD [97]
Annual Baltimore overdose deaths (*D*)	463	Heroin-related overdose deaths in first three quarters of 2016 extrapolated to full year	DHMH [1, 47]
Estimated value per overdose death averted (*V*)	$503,869	Calculated by authors using the variables below.	
Average years until retirement (*N*)	30	Assuming retirement age of 65	Genberg et al. [49]
Median wage for Baltimore City (*W*)	$25,707		Census Bureau [98]
Discount rate (*r*)	3%		Andresen and Boyd [44]

Most likely, this method underestimates the facility’s impact, since this method only estimates averted overdose deaths within 500 m of the SIF, though the facility would also reduce overdose outside a 500-m radius.

### Overdose morbidity benefits

Overdoses require emergency medical assistance, even when they are not life threatening. Evaluations of Sydney’s MSIC show that by managing overdose events on-site, the SIF reduces ambulance calls, emergency room visits, and hospital stays for overdose-related morbidity [12]. No previous SIF cost-benefit evaluations have included overdose morbidity in their analyses, but MSIC provides sufficient data to estimate the magnitude of a SIF’s impact.

In Baltimore, ambulances are called to the scene of roughly half of all nonfatal overdoses [50]. By contrast, almost all overdoses in MSIC, Sydney’s SIF, were handled by on-site medical staff and did not result in ambulance calls [14]. We estimate cost savings of averted ambulance calls for a SIF in Baltimore according to the following model:

$$ {S}_{\mathrm{a}}= Io\left({c}_o-{c}_i\right) A $$

where S_a_ is the annual savings due to the SIF reducing ambulance calls for overdose, *I* is the annual number of injections in the SIF, *o* is the per-injection rate of overdose, *c*_*o*_ and *c*_*i*_ are the rates of overdose ambulance calls outside and inside the SIF, respectively, and *A* is the average cost of an overdose ambulance call. The values and sources for these variables are given in Table 5.

**Table 5:** Values, notes, and sources for variables used to predict savings from overdose-related ambulance calls

Variable	Value	Note	Source
Total annual injections in the SIF (*I*)	180,000	Based on Insite capacity and use	Milloy et al. [45]
Percent of injections resulting in overdose (*o*)	0.133%	Insite’s rate used as conservative estimate, since Baltimore has a higher overdose rate than Vancouver	Kerr et al. [99], Kerr et al. [16], Milloy et al. [45], Astemborski and Mehta [100]
Rate of overdose resulting in ambulance call (*c*_o_)	46%		Pollini et al. [50]
Rate of SIF overdose ambulance call (*c*_*i*_)	0.79%	For MSIC	KPMG [14]
Cost of overdose ambulance call (*A*)	$750	For Baltimore County	Baltimore County [101]

Emergency response personnel often transport overdose victims to the emergency room for treatment. One Baltimore study found that 33% of PWID reported being taken to the ER for their latest overdose [50]. By contrast, overdoses in SIFs lead to emergency room treatment in less than 1% of cases [14]. With a single Baltimore ER visit averaging over $1,300, SIFs reduce medical costs significantly by keeping PWID out of emergency rooms for overdose. We calculate the savings according to the following:

$$ {S}_{\mathrm{er}}= Io\left({t}_o-{t}_i\right) F $$

where *S*_er_ is the annual savings due to the SIF reducing emergency room visits for overdose, *I* is the annual number of injections in the SIF, *o* is the rate of nonfatal overdose, *t*_*o*_ and *t*_*i*_ are the rates of ER visit for overdose when the overdose occurs outside and inside the SIF, respectively, and *F* is the average cost of an overdose emergency room visit. The values and sources for these variables are given in Table 6.

**Table 6:** Values, notes, and sources for variables used to predict savings from overdose-related emergency room visits

Variable	Value	Note	Source
Total annual injections in the SIF (*I*)	180,000	Based on Insite capacity and use	Milloy et al. [45]
Percent of injections resulting in overdose (*o*)	0.133%	Insite’s rate used as conservative estimate, since Baltimore has a higher overdose rate than Vancouver	Kerr et al. [99], Kerr et al. [16], Milloy et al. [45], Astemborski and Mehta [100]
Rate of overdose resulting in emergency room visit (*t*_o_)	33%		Pollini et al. [50]
Rate of SIF overdose emergency room visit (*t*_*i*_)	0.79%	Ambulance call rate for MSIC, an upper bound for emergency room visits	KPMG [14]
Cost of overdose emergency room visit (*F*)	$1,364	Average Baltimore City emergency room visit cost	Rienzi [102]

Overdose victims are occasionally hospitalized for treatment. In Baltimore, 12% of PWID who overdosed reported being hospitalized, while less than 1% of SIF overdoses lead to hospitalization [14, 50]. With one day in a Baltimore hospital averaging $2,500, SIFs reduce medical costs significantly by keeping PWID out of the hospital for overdose. We calculate the savings according to the following:

$$ {S}_{\mathrm{h}}= Io\left({a}_{\mathrm{o}}-{a}_{\mathrm{i}}\right) E $$

where *S*_h_ is the annual savings due to the SIF reducing hospitalization for overdose, *I* is the annual number of injections in the SIF, *o* is the rate of nonfatal overdose, *a*_o_ and *a*_i_ are the rates of hospitalization for overdose when the overdose occurs outside and inside the SIF, respectively, and *E* is the average expense of an overdose hospital stay. The values and sources for these variables are given in Table 7.

**Table 7:** Values, notes, and sources for variables used to predict savings from overdose-related hospitalizations

Variable	Value	Note	Source
Total annual injections in the SIF (*I*)	180,000	Based on Insite capacity and use	Milloy et al. [45]
Percent of injections resulting in overdose (*o*)	0.133%	Insite’s rate used as conservative estimate, since Baltimore has a higher overdose rate than Vancouver	Kerr et al. [99], Kerr et al. [16], Milloy et al. [45], Astemborski and Mehta [100]
Rate of overdose resulting in hospitalization (*a*_o_)	12%		Pollini et al. [50]
Rate of SIF overdose hospitalization (*a*_*i*_)	0.79%	Ambulance call rate for MSIC, an upper bound for hospitalizations	KPMG [14]
Cost of overdose hospitalization (*E*)	$2500	Average hospital day cost for Maryland	Pfuntner [103]

### Medication-assisted treatment benefits

Many PWID who are unable to quit using illicit opioids through traditional abstinence-based treatment programs are successful using methadone or buprenorphine maintenance as part of medication-assisted treatment (MAT) [51]. MAT not only reduces the crime and health care costs of PWID by helping a significant portion quit injecting drugs but also decreases drug use, crime, and health costs among the patients who do relapse [52, 53]. Wood et al. [15, 22] and MSIC [12] show that both Insite and Sydney’s MSIC refer many SIF clients to treatment, increasing treatment uptake. Irwin et al. [31] find a single SIF’s impact on treatment uptake to be significant, estimating that a SIF in San Francisco would bring 110 patients into MAT every year.

We estimate that by referring clients to MAT, a SIF would produce annual health care and crime savings equal to *S*_MAT_:

$$ {S}_{\mathrm{MAT}}= N r\; f\left( b-1\right) T $$

where *N* is the number of PWID who use the SIF, *r* is the percent of SIF clients who have been shown to access treatment as a result of SIF referrals, *f* is a conservative 50% estimate for retention in MAT, *b* is the average cost-benefit ratio studies have found for MAT, and *T* is the annual cost of treatment. Table 8 shows the values and sources for each variable.

**Table 8:** Sources for variables used to predict savings from medication-assisted treatment referrals

Variable	Value	Note	Source
Number of SIF clients (*N*)	2100	Approximate monthly unique Insite injection room clients	Maynard [33]
Percent of SIF users who access MAT as a result of SIF referrals (*r*)	5.78%	From MSIC	MSIC [12]
Treatment retention factor (*f*)	50%	General retention rate estimated at 60–90%	CSAM [104]
Cost-benefit ratio for MAT (*b*)	4.5	Conservative: average of low estimates	Cartwright [51], Gerstein [105], Health Canada [32], Harris et al. [52], CHPDM [53]
Average cost of 1 year of MAT (*T*)	$4000		Schwartz et al. [106]

The SIF’s success in referring PWID to MAT depends on the pre-existing local prevalence of MAT uptake, location and availability of MAT slots, and other neighborhood-level factors. As a result, we acknowledge that the 5.8% increase found for Sydney’s MSIC may differ significantly from the actual referral rate for a SIF in Baltimore.

## Results

### Overall cost-benefit ratio

Our analysis finds a total benefit of $7.77 million and a total cost of $1.79 million, yielding a cost-benefit ratio of $4.35 saved for every dollar spent. Net savings are $5.98 million. We present the sensitivity analysis results for each outcome in Table 9, showing both financial and health results for the base, low, and high cases. Table 10 shows the impact of the sensitivity analysis for each key variable on the overall cost-benefit ratio and net savings.

**Table 9:** Summary of sensitivity analysis impact for individual components

Outcome	Dollar value ($ million)			Health indicator value			
	Base case	Low case	High case	Base case	Low case	High case	Unit
Total cost	1.79	2.60	0.98				
HIV	1.50	0.75	2.25	3.7	1.8	5.5	Cases
HCV	1.44	0.72	2.17	21	11	32	Cases
SSTI	0.93	0.47	1.40	374	187	561	Hospital days
Overdose deaths	3.00	1.50	4.50	5.9	3.0	8.9	Deaths
OD ambulance calls	0.08	0.04	0.12	108	54	162	Calls
OD ER visits	0.11	0.05	0.16	78	39	117	ER visits
OD hospitalizations	0.07	0.03	0.10	27	13	40	Hospitalizations
MAT	0.64	0.32	0.96	121	61	182	New patients

**Table 10:** Summary of sensitivity analysis impact on overall results

Variable tested	Cost-benefit ratio			Net savings ($ million)		
	Base case	Low case	High case	Base case	Low case	High case
Operating cost	4.35	2.99	7.96	5.98	5.17	6.79
Syringe sharing rate	4.35	3.52	5.17	5.98	4.51	7.46
SSTI rate	4.35	4.09	4.61	5.98	5.52	6.45
Overdose death rate	4.35	3.51	5.19	5.98	4.48	7.48
Nonfatal OD rate	4.35	4.28	4.42	5.98	5.86	6.11
MAT referral rate	4.35	4.17	4.53	5.98	5.66	6.30

### Cost of the facility

Our estimate of the total annual cost is $1.79 million, which includes $1.62 million in operating costs and $170,000 in annualized upfront costs. In our sensitivity analysis, raising the operating cost by 50% increased the total cost to $2.6 million, lowering the cost-benefit ratio from 4.35 to 2.99 and net annual savings from $5.98 million to $5.17 million. Lowering the operating cost by 50% resulted in a total cost of $980,000, raising the cost-benefit ratio to 7.96 and net savings to $6.79 million.

### HIV and HCV benefits

We estimate that a SIF would prevent an average of 3.7 HIV and 21 HCV cases per year, translating to annual savings of $1.50 million and $1.44 million, respectively.

We conducted a sensitivity analysis on the syringe sharing rate. Increasing the rate by 50%, from 2.8 to 4.2%, raises averted infections to 5.5 for HIV and 32 for HCV and savings to $2.25 million for HIV and $2.17 million for HCV. As a result, the overall cost-benefit ratio for the SIF increases from 4.35 to 5.17 and net savings increase from $5.98 million to $6.45 million. Decreasing the sharing rate by 50%, from 2.8 to 1.4%, lowers averted infections to 1.8 for HIV and 11 for HCV, reducing HIV savings to $750,000 and HCV savings to $720,000. In this scenario, the overall cost-benefit ratio declines to 3.52 and net savings fall to $4.51 million.

### Skin and soft-tissue infection benefits

We estimate that SIF SSTI care will reduce total PWID hospital stays for SSTI by 374 days per year, which translates to annual savings of roughly $930,000.

We conducted a sensitivity analysis on the SSTI hospitalization rate. Increasing the rate by 50% raises averted hospital days to 561 and savings to $1.40 million. As a result, the overall cost-benefit ratio for the SIF increases from 4.35 to 4.61 and net annual savings rise from $5.98 million to $6.45 million. Decreasing the rate by 50% lowers averted hospital days to 187 and reduces savings to $470,000. In this scenario, the overall cost-benefit ratio declines to 4.09 and net savings fall to $5.52 million.

### Overdose mortality benefits

We estimate that SIF overdose prevention will save an average of 5.9 lives per year, which translates to $3.00 million in savings for society.

We conducted a sensitivity analysis of drug overdose deaths in the neighborhood around the facility, since deaths fluctuate from year to year. Increasing the total by 50% raises estimated lives saved to 8.9 and financial savings to $4.50 million. This raises the overall cost-benefit ratio for the SIF from 4.35 to 5.19 and net savings from $5.98 million to $7.48 million. Lowering the neighborhood deaths by 50% would reduce estimated lives saved to 3.0 and financial savings to $1.50 million, for an overall cost-benefit ratio of 3.51 and net savings of $4.48 million.

### Overdose morbidity benefits

We estimate that the SIF will also prevent 108 ambulance calls, 78 emergency room visits, and 27 hospitalizations for nonfatal overdose, which translates to $81,000, $110,000, and $67,000 in medical savings, respectively.

We conducted a sensitivity analysis on the nonfatal overdose rate, since it is not well documented for Baltimore. Increasing the rate 50% raises the benefits to 162 ambulance calls, 117 ER visits, and 40 hospitalizations, for savings of $120,000, $160,000, and $100,000, respectively. This higher rate would raise the overall cost-benefit ratio for the SIF from 4.35 to 4.42 and net savings from $5.98 to $6.11 million. Lowering the rate by 50% would reduce the benefits to 54 ambulance calls, 39 ER visits, and 13 hospitalizations, lowering the savings to $40,000, $50,000, and $30,000, respectively. This lower rate would reduce the SIF’s overall cost-benefit ratio to 4.28 and net savings to $5.86 million.

### Medication-assisted treatment benefits

We estimate that 121 PWID will enter MAT as a result of the SIF, translating into $640,000 in benefits for society.

We conducted a sensitivity analysis of the referral rate for MAT. Raising the rate by 50%, from 5.78 to 8.67%, would increase new people in treatment from 121 to 182 and financial savings to $960,000. This would increase the overall cost-benefit ratio from 4.35 to 4.53 and net annual savings from $5.98 to $6.30 million. Lowering the rate by 50%, to 2.89%, would reduce new people in treatment to 61 and financial savings to $320,000, for an overall cost-benefit ratio of 4.17 and net savings of $5.66 million.

## Discussion

Our analysis finds a significantly favorable cost-benefit ratio and net benefits in all scenarios for a SIF in Baltimore, MD. Our base case scenario predicts that every dollar spent would return $4.35 in savings. We estimate that a single, 13-booth facility would generate annual net savings of $5.98 million, which is equivalent to 28% of the city health department’s entire budget for harm reduction and disease prevention [54]. The study predicts that a SIF would prevent 5.9 overdose deaths per year.

Compared to Irwin et al.’s [31] cost-benefit analysis of a SIF in San Francisco, our study estimates the cost-benefit ratio for a Baltimore SIF to be 87% higher (4.35 versus 2.33) and net savings to be 71% higher ($6.0 million versus $3.5 million). A Baltimore SIF would have lower costs, lower benefits from SSTI prevention, similar benefits related to HIV, HCV, and MAT, and much higher benefits related to overdose deaths. Our study also incorporates additional outcomes, demonstrating that a SIF could generate sizeable benefits by preventing ambulance calls, emergency room visits, and hospital stays related to nonfatal overdose.

The most significant difference between the San Francisco and Baltimore studies relates to the SIF’s impact on overdose deaths. We predict 5.9 lives saved per year in Baltimore, compared to 0.24 lives in San Francisco [31]. This difference stems primarily from the much higher overdose death rate in Baltimore. While both cities have roughly 20,000 PWID, Baltimore has more than 20 times more heroin-related overdose deaths. We also use a more advanced methodology—mapping the concentration of overdose deaths—to estimate this outcome.

The SIF’s impact on overdose prevention would complement the Baltimore City Health Department’s extensive efforts to prevent overdose through trainings and naloxone distribution in community, treatment, and corrections settings. The city has trained over 17,500 Baltimore residents in overdose prevention, including use of the overdose reversal drug naloxone [55]. A SIF would ensure that when PWID overdose, they do so in the presence of staff trained to administer naloxone. In addition, a SIF would prevent overdose deaths outside the facility because SIF staff provide PWID with safer injecting education, stressing the importance of injecting where naloxone is available.

Our results also suggest that a SIF would become a key component of Baltimore’s continued efforts to reduce viral infections among PWID. Preventing four HIV and 21 HCV infections every year would reduce total incidence of both HIV and HCV by roughly 5%. The SIF would allow service providers to locate PWID, test them for viral infection, refer them for HIV and HCV treatment, and retain them in treatment. It thus addresses all four aspects of the 2017 HIV prevention strategy of the National Institute on Drug Abuse: “seeking, testing, treating, and retaining” PWID and other populations in need of HIV care [56].

Our estimate that a SIF would save close to a million dollars per year in SSTI hospital costs shows the benefits of removing a small population of “frequent fliers” from emergency rooms and hospitals. Still, since San Francisco has both a more serious SSTI problem due to the prevalence of black tar heroin and higher hospital costs, this area of benefits is smaller for Baltimore.

Our estimate of 121 PWID entering MAT in Baltimore is similar to Irwin et al.’s [31] estimate of 110 PWID in San Francisco. However, in both cities, the actual number will depend on the existing ease of MAT access, as well as the efforts by SIF staff to refer PWID to treatment. Baltimore can maximize these benefits by increasing funding to MAT programs, making treatment referrals a priority for SIF staff, and establishing the SIF near existing treatment providers for easy referral and follow-up.

Our sensitivity analysis illustrates that the SIF’s operating cost has a significant impact on the overall cost-benefit ratio, though less of an impact on net savings. While we used a conservatively high cost estimate, strategic staffing, location, and procedural decisions by both SIF executives and local government officials could reduce costs and further increase the net benefits. Cost-effectiveness in Baltimore would be significantly higher largely because Baltimore has lower real estate values, salaries, cost of living, and cost of doing business [31].

There are a number of lessons from the initial operations of Insite which could inform the overall costs associated with a SIF in Baltimore. For example, Health Canada’s protocols required Insite to call an ambulance for every overdose incident, resulting in unnecessary costs given the ability to reverse overdose at Insite [57]. We recommend that the Baltimore City Health Department work with a local SIF, with extensive peer involvement, to consider the health, social, and economic impact of any such protocols.

The continuum of care provided at the SIF has important implications for its impact. An integrated SIF model would co-locate detoxification, treatment, medical care, mental health care, housing, employment, government benefits, and legal services. Such a model would facilitate service uptake for a population that faces a number of barriers in accessing services.

We should note that it is difficult to ascertain who exactly would ultimately receive the savings documented in this study. Savings from the HIV, HCV, SSTI, and nonfatal overdose outcomes all accrue to the health care system, but the real beneficiaries are difficult to pin down. Holtgrave [58] and Mehta [6] estimate that the public sector bears the greatest share of HIV treatment costs, in particular Medicaid. Whether PWID have private insurance, Medicare/Medicaid, or no insurance, the savings ultimately reach federal, state, and local taxpayers, as well as everyone who pays health care premiums and hospital bills. MAT savings are split between medical care and reduced crime committed to get money to buy drugs. Overdose death savings represent value to the overall local economy from that person’s future contributions.

### Limitations

This cost-benefit analysis faces a number of limitations.

First, this study does not tackle the political, legal, and social barriers confronting the efforts to establish a SIF in Baltimore. In spring 2017, a second attempt to authorize safe consumption spaces in Maryland failed in the Maryland State Assembly. This effort faces opposition concerns similar to SIF campaigns in other cities, including fears of “enabling” drug use, “Not In My Back Yard,” and potential legal vulnerability to prosecution under federal drug statutes [59–61]. It also faces more unique challenges—while the opiate epidemic’s recent damage to white, middle-class communities has grabbed media attention, Baltimore’s heroin crisis is decades old and fails to generate the same political capital for action because it primarily impacts lower-income African-American communities [62].

To address these issues, advocates have formed a coalition of public health practitioners, current and former drug users, community organizers, and academics. Over the past year, the coalition has been meeting with the local health department, social service providers, drug users, politicians, and community leaders. In addition to garnering local and state political support, a Baltimore SIF campaign will only be successful if it involves the affected communities and elevates their voices.

Our study’s estimates of health and economic outcomes also face limitations. Without specific plans for a facility, some variables are difficult to estimate. Since there are no actual regulations, guidelines, or actual physical plans for a SIF in Baltimore, we can only make a conservative guess at facility cost. Once regulations are established and plans for construction and operation have been created, an updated cost analysis should be performed. Similarly, the SIF’s success at referring PWID to treatment would depend on staffing decisions, the protocol for treatment referrals, and the convenience and availability of effective treatment options.

In addition, our models are difficult to verify because a number of important health indicators are not well documented for Baltimore’s PWID population. For example, researchers have noted that resources have not been devoted to accurately measuring the Baltimore PWID population’s HCV prevalence, much less the HCV incidence or the impact of needle sharing [63]. Also, available data conflicts on the prevalence of SSTI and rates of SSTI hospitalization among PWID. Other variables, from the average number of needle-sharing partners to the rate of ambulance calls to nonfatal overdose, are based on a single study and should be corroborated.

The study’s accuracy would also benefit from specific cost information. The costs of HIV and HCV care, SSTI hospitalization, medication-assisted treatment, and overdose-related ambulance calls, emergency room visits, and hospital stays have all been approximated using figures for the general population. We consider all of these to be underestimates of the actual costs, since PWID tend to require more services and supervision [64].

There are also some potential interaction effects that are beyond the scope of this study. For example, our HIV and HCV models do not account for PWID becoming infected or transmitting the viruses to others through sexual contact. Our models also do not account for interaction effects between HIV and HCV infection or between viral infection and SSTI. While these effects would likely have a minor impact on our overall findings, if relevant data becomes available, our analysis should be updated accordingly.

Finally, the impact of the SIF will depend on how well the SIF and co-located service providers align with the unique features of Baltimore’s population of PWID. Studies have shown that the effectiveness of harm reduction programs depends on their consideration of ethnicity, gender, age, homelessness, inequality, social networks, drug markets, and other demographic and social factors [65–70]. We have used the best local health data available to tailor our analysis to Baltimore’s unique risk factors and social environment. However, the ultimate impact of a SIF in Baltimore will depend on how well the facility adapts to this environment by studying, consulting, and collaborating with the local PWID population [71–73].

## Conclusions

Despite the present study’s limitations, it demonstrates that a SIF in Baltimore would bring significant cost savings and public health benefits to the city. A single 13-booth SIF facility in Baltimore City modeled on Insite in Vancouver would generate medical and economic savings of roughly $7.77 million per year. At a total cost of $1.79 million per year, every dollar spent would generate an estimated $4.35 in savings. To put the $5.98 net annual savings for a single SIF in perspective, they equal 28% of the Baltimore City Health Department’s budget for harm reduction and disease prevention.

In terms of health outcomes, we estimate that every year, a SIF would prevent 3.7 HIV infections, 21 HCV infections, 374 days in the hospital for skin and soft-tissue infection, 5.9 overdose deaths, 108 overdose ambulance calls, 78 overdose emergency room visits, and 27 overdose-related hospitalizations, while bringing an additional 121 PWID into treatment.

We recommend that the city avoid excessive regulation of a SIF and maximize the linkages to services for the PWID population. We also recommend that researchers carefully track health indicators and medical costs associated with the PWID population before and after establishing a SIF in order to evaluate the facility’s benefits.

SIFs provide other important benefits in addition to those quantified in this study. They decrease public injection, prevent physical and sexual violence against PWID, and reduce syringe littering [38, 74–76]. They facilitate research to better understand the PWID population [77]. Lastly, they allow social service providers to harness the power of PWID peer networks and bring important programs to the hard-to-reach PWID population [78–80].

Establishing a SIF in Baltimore would bring a number of well-established medical, financial, and societal benefits. We do not believe that health initiatives like SIFs should be judged purely on financial terms. However, we hope that this cost-benefit analysis provides a helpful starting point to assess the potential impact on Baltimore of a supervised injection facility.

## Abbreviations

DHMH: Department of Health and Mental Hygiene (Maryland)
HCV: Hepatitis C virus
HIV: Human immunodeficiency virus
MAT: Medication-assisted treatment
MSIC: Medically Supervised Injecting Centre (SIF in Sydney)
OD: Overdose
PWID: People who inject drugs
SIF: Supervised injection facility
SSTI: Skin and soft-tissue infection
